# Examining a Ripple Effect: Do Spouses' Behavior Changes Predict Each Other's Weight Loss?

**DOI:** 10.1155/2013/297268

**Published:** 2013-09-05

**Authors:** Anna E. Schierberl Scherr, Kimberly J. McClure Brenchley, Amy A. Gorin

**Affiliations:** ^1^Department of Psychology, University of Connecticut, 406 Babbidge Road, Unit 1020, Storrs, CT 06269, USA; ^2^Department of Psychology, St. John Fisher College, 3690 East Avenue, Rochester, NY 14618, USA; ^3^Department of Psychology, Center for Health, Intervention and Prevention, University of Connecticut, 2006 Hillside Road, Unit 1248, Storrs, CT 06269, USA

## Abstract

*Background*. Including spouses in obesity treatment has been found to promote weight loss. We assessed whether spouses' diet and activity changes impacted each other's weight loss when both members attended an active weight loss program (TOGETHER) or only the primary participant attended treatment (ALONE). *Methods*. Heterosexual couples (*N* = 132) enrolled in an 18-month randomized controlled weight loss trial were weighed and completed measures of dietary intake and physical activity at baseline and 6 months. We conducted dyadic data analyses using the Actor-Partner Interdependence Model. *Results*. Participants' weight loss was not predicted by their partners' behavior changes. However, partners' weight loss was predicted by their participants' changes in calorie and fat intake. When partners were coupled with a participant who did not reduce their own calorie and fat intake as much, these partners had higher weight loss when treated in the TOGETHER group but lower weight loss when they were untreated in the ALONE group. There were no reciprocal effects found with physical activity changes. *Conclusions*. Direct treatment had the greatest impact on participants and partners who were treated. Untreated partners' weight losses were positively impacted by their spouses' dietary changes, suggesting a ripple effect from treated spouses to their untreated partners.

## 1. Introduction

 Enhancing weight loss maintenance is an imperative in obesity treatment, and social networks are poised to be important facilitators of this process. A growing body of research suggests that romantic partnerships exert an influence on obesity and therefore may be an important network to intervene upon [1–4]. Married people are generally heavier, and weight gain, decreased physical activity, and poor diet changes after marriage are common [4–8]. Spouses tend to gain weight during the first few years of marriage [9, 10], and increased duration of cohabitation with romantic partners is associated with obesity [7].

 Why married individuals tend to share an obesity risk is less understood. The mechanisms of assortative mating and shared home environment are potential explanations [11–13]. Assortative mating suggests that individuals select romantic partners with similar behaviors and body types. Thus, from the start of relationships, couples share an obesity status and behaviors that perpetuate this condition [12]. Similarly, the shared home environment mechanism suggests that spouses share an obesity risk, but instead the risk occurs as a result of their shared household, finances, and social networks [13]. Concordance data provide additional support that something unique about spouses sharing a home environment confers an obesity risk. Married couples have similar weight measurements (i.e., BMI, fat distribution, waist circumference, and waist/hip circumference ratio) [1, 11, 14–19], as well as similar diet and physical activity behaviors such as fat, fruit, vegetable, egg and milk consumption and exercise frequency and distance [11, 17, 20, 21]. 

 Behavioral weight-loss treatment (BWL) is well positioned to harness spousal similarities to reverse the often obesogenic effects of marriage. BWL, the gold standard treatment for overweight to moderately obese individuals, teaches behavioral strategies to facilitate change in diet and physical activity behaviors [22]. By emphasizing environmental antecedents and consequences of eating and exercise, behavioral theory suggests that spouses should have an impact on one another's behaviors and ultimate weight loss and maintenance [22]. Accordingly, untreated spouses have been found to lose nearly 3% of their body weight when the other spouse enters a weight-loss program [2]. Benefits have also been found when spouses were included directly in treatment; they achieved modest weight losses for up to 18 months after treatment when incorporated into standard behavioral programs [23, 24]. The positive effects, however, diminished over time [23, 24]. This evidence suggests that spousal relationships can be used to promote weight loss, but understanding how partners impact each other's weight loss may be a necessary step to ensure that spouses are being included in a way that fosters long-term change. 

 This secondary analysis examined the impact of spouses' diet and activity changes on one another's 6-month weight loss outcomes from the Lifestyle Eating and Activity Program (LEAP), a randomized controlled trial comparing a comprehensive weight-loss program that targeted an individual's behavior and his or her physical and social home environment to standard behavioral weight-loss treatment. Gorin et al. (2013) reported that in the primary LEAP trial there was a significant effect of the home environment intervention group on initial weight loss (*p* < .05) [25]. Participants in the home environment intervention had significantly greater weight loss than participants in standard behavioral weight-loss treatment (*p* = .017) at 6 months, but at 18 months this difference was no longer observed (*p* = .19). Gender moderated the treatment response at both 6 months and 18 months (Group x Gender at 6 months, *p* = .011; at 18 months, *p* = .006). Females lost significantly more weight in the home environment intervention at 6 and 18 months, whereas males lost the same amount of weight in both groups at 6 months and lost more weight in standard behavioral weight-loss treatment at 18 months.

 A key component of the home environment intervention group in the LEAP trial was the inclusion of support partners. Conversely, in the standard behavioral weight loss arm, only the primary participant attended treatment. In the current study, we limited our analyses to LEAP's married couples and compared couples in which both members were randomly assigned to participate together in the home environment intervention group (hereafter referred to as the “TOGETHER” group) to couples in which only the primary participant attended standard behavioral weight-loss treatment (hereafter referred to as the “ALONE” group). We sought to answer four primary questions: (1) Are participant's behavior changes related to their own weight loss? (2) Are spouse's behavior changes related to the spouse's own weight loss? (3) Are participant's behavior changes related to the spouse's weight loss? (4) Are spouse's behavior changes related to the participant's weight loss? Because couples shared a home environment and were concordant for intervention targets we hypothesized that the behavior changes made by couples in the TOGETHER condition would demonstrate reciprocal impact for weight loss as compared to those in the ALONE condition. 

## 2. Materials and Methods

### 2.1. Participants

Two hundred and one overweight and obese individuals were included in the LEAP trial from which a subsample of 132 married, heterosexual couples were drawn for this secondary analysis. We focused on this group because we were interested in the unique relationship between marriage and weight loss, and reciprocal impacts among the other dyad pairings (e.g., parent-adult child, roommates, etc.) may not follow a similar pattern. Individuals were eligible for LEAP if they met the following criteria: were aged 21–70 years old, have a body mass index (BMI) between 25 and 50 kg/m^2^, and have a household member willing to participate in the study as a support partner. Partners had to be interested in weight loss and have a BMI between 25–50 kg/m^2^. Participants and spouses were excluded from participating if they reported chest pain during periods of activity or rest, a heart condition, loss of consciousness, being unable to walk two blocks without stopping, current participation in another weight-loss program and/or taking weight loss medication, recent weight loss of 10 lbs. within three months of screening, recent or current pregnancy or nursing, planning on becoming pregnant in the next two years, or any condition that in the judgment of the research team made it unlikely the individual would complete the study protocol (i.e., plans to move out of the area, substance abuse, significant psychiatric problems, dementia, and terminal illness). Individuals endorsing joint problems, prescription medication usage, or other medical conditions that could limit exercise were required to obtain written physician consent to participate. The study was approved by The Miriam Hospital's Institutional Review Board.

### 2.2. Intervention

 Treatment across conditions was modeled after several recent trials, including Look AHEAD and PRIDE, and was designed to produce a weight loss of 7–10% of body weight [26, 27]. All participants were placed on a standard caloric and fat restricted diet (e.g., 1200–1800 kcals/day and 30% fat, depending on initial weight) and given sample meal plans and a calorie guidebook to help them meet their goals. Participants were instructed to gradually increase their physical activity until they were achieving >200 minutes of moderate intensity physical activity per week. Treatment also included training in core behavioral skills including self-monitoring, stimulus control, problem solving, goal setting, cognitive restructuring, and relapse prevention. Treatment focus shifted to weight loss maintenance in the latter months of the program. Groups met weekly for six months followed by biweekly meetings for 12 months and were led by interventionists with a master's or doctoral degree in nutrition, exercise physiology, or behavioral psychology and experience providing weight-loss treatment. 

The primary difference between the treatment conditions was that ALONE focused on the individual participant, while TOGETHER targeted the individual and their spouse plus physical and social cues within their homes. Physical environment manipulations in the TOGETHER condition aimed to cue healthy behavior choices (e.g., motivational posters, treadmill, scale, TV, and serving-size appropriate dishware). Social manipulations in the TOGETHER intervention included partner involvement (i.e., spousal involvement in the case of this study) in an effort to create a positive model in the home. TOGETHER spouses were encouraged to set a 7–10% weight loss goal, attend all weight loss groups, and use the identical behavioral tools as the participants, including daily self-monitoring. To encourage participants and spouses to work together, various campaigns related to eating and exercise behaviors were held throughout the program; for example, participant-spouse pairs competed against other pairs to receive small prizes. 

In the ALONE condition, participants were encouraged to share information with spouses congruent with standard behavioral treatment. Spouses were untreated and were not given any instructions about supporting their spouses' weight loss. They attended a one-hour Weight Loss 101 session during which standard behavioral approaches to weight loss were shared. Specifically, spouses were instructed about principles of the energy balance and how to self-regulate and self-monitor, although they were not required to set behavioral goals. 

### 2.3. Data Collections

 The following measures were completed by participants and spouses at baseline and six months. Basic demographic information was collected at baseline.

#### 2.3.1. Weight and Height

Weight was measured on a calibrated digital scale (Tanita BWB 800), with individuals in light-weight clothing and shoes removed. Height was measured on a calibrated, wall-mounted stadiometer. BMI was calculated as kg/m^2^.

#### 2.3.2. Dietary Intake

Dietary intake was assessed using the self-report Block Food Frequency Questionnaire [28]. We examined daily caloric intake and daily fat intake over the six-month recall period. The Block has been found to correspond with dietary records and has been validated against three-day records [28, 29]. 

#### 2.3.3. Physical Activity

Energy expenditure was assessed using the Paffenbarger Activity Questionnaire (PAQ) [30]. We examined total minutes of physical activity per week over the one-week recall period. The PAQ has high test-retest reliability and is significantly correlated with measures of cardiovascular fitness [31, 32].

### 2.4. Data Analysis

**Figure 1:**
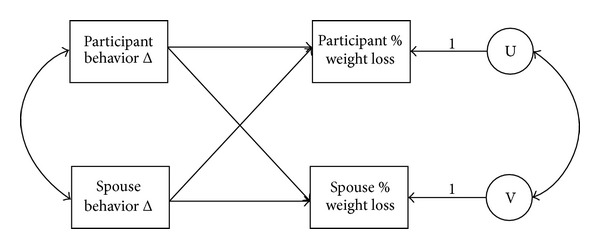
Basic APIM: conceptual model.

The data were analyzed with structural equation modeling using the Actor-Partner Interdependence Model (APIM) in IBM SPSS Amos 20 [33, 34]. The APIM allows simultaneous examination of each dyad member's influence on their own outcomes as well as each member's influence on their partner's outcomes, resulting in four unique effects. For example, in the current study, our four primary research questions can be examined simultaneously by using the APIM. See Figure 1 for a conceptual model showing each of the four effects. Because data from each dyad member are interdependent, each member's predictor variables were allowed to correlate, as were the error variances of their outcome variables (as shown in Figure 1). A separate APIM was estimated for each behavior change: total daily calorie intake, percent fat in diet, and total exercise minutes. 

 Within each APIM, the effect of each dyad member's behavior change on their weight loss was controlled for by their baseline behavior. The base APIM model examining the four effects was also extended to test treatment group as a moderator. In order to accomplish this, treatment group was added as a main effect on each dyad member's weight loss, and interaction terms were created between group and each dyad member's behavior change. Because there were no significant participant or spouse differences between groups for age, gender, ethnicity, and BMI (*ps* > .05), these variables were not added as covariates in favor of a more parsimonious model. 

## 3. Results

 This secondary data analysis included 132 heterosexual married couples; the ALONE group contained 68 couples and the TOGETHER group contained 64 couples. Of the 132 couples, women were the primary participants in 97 of the couples, and men were the primary participants in 35. Mean BMI for the entire sample was 34.2 kg/m^2^. There were no significant participant or spouse differences between groups for age, gender, ethnicity, or BMI (*ps* > .05). Demographic characteristics for participants and spouses can be seen in Table 1. Baseline and change characteristics for all variables can be seen in Table 2. Correlations between spouses for BMI and behaviors at baseline and 6 months can be seen in Table 3. All further results represent effects found over and above the influence of treatment group.

### 3.1. Energy Intake

 The relationship between six-month changes in energy intake (kcal/day) and weight loss was examined using APIM. Two of our primary research questions pertained to the participant's weight loss: whether participants' changes in energy intake are associated with their own weight loss and whether their spouses' changes in energy intake are associated with the participants' weight loss. Neither effect was significant in our model. Instead, treatment group and baseline energy intake emerged as significant covariates for the participant. Higher energy intake at baseline was associated with lower percent weight loss over six months, *β* = .24, *p* = .026. 

 We also had two primary research questions that pertained to the spouse's weight loss: whether spouses' changes in energy intake predict their own weight loss and whether participants' changes in energy intake predict their spouses' weight loss. In terms of whether spouses' changes in energy intake over six months predict their own weight loss, this effect was significant, *β* = .41, *p* = .005. Spouses who decreased their energy intake had higher percent weight loss. 

**Figure 2:**
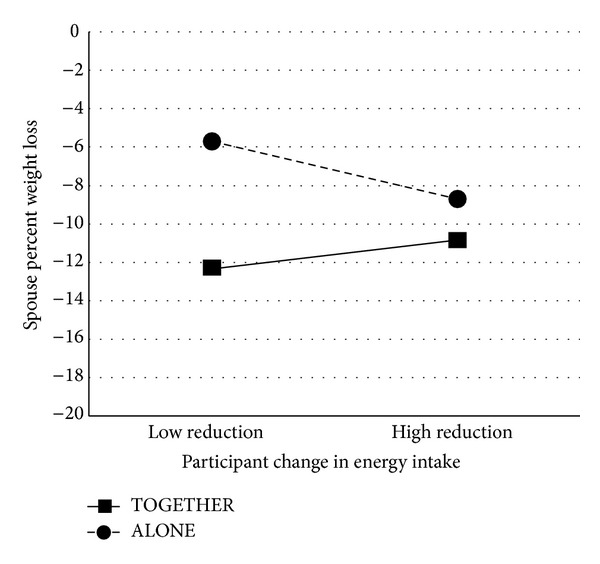
Interaction between treatment group and participants' energy intake change when predicting spousal weight loss. High and low calorie reduction are defined as ±1 SD from the mean reduction score of 546.62 kcal. “High reduction” thus corresponds to a reduction of 1295.14 kcal, and “low reduction” corresponds to a gain of 201.90 kcal.

 In terms of whether participants' changes in energy intake predict their spouses' weight loss, we found that the effect depended on which treatment group the couple participated in, *β* = .19, *p* = .046 (see Figure 2). A significant interaction between treatment group and participant behavior change indicated that spouses in the TOGETHER and ALONE groups lost similar percentage of weight loss to one another when their participants reduced their own caloric intake over six months to a greater degree. However, when participants did not decrease their caloric intake as much over the six months, their spouses had higher percent weight loss when they were treated in the TOGETHER group. For spouses left untreated in the ALONE group, they had lower percent weight loss when coupled with a participant who did not decrease their own caloric intake as much over the six months.

### 3.2. Fat Intake

 The relationship between six-month changes in fat intake (% kcal/day) and weight loss was examined using APIM. Similar to the energy intake results, our two primary research questions pertaining to the separate influences of the participant's and spouse's fat intake changes on the participant's weight loss did not emerge as significant. Baseline fat intake emerged as a significant covariate, *β* = .24, *p* = .028. Specifically, higher fat intake at baseline was associated with lower percent weight loss over six months. 

 For our two primary research questions that pertained to the spouse's weight loss, similar results to that of energy intake change also emerged. In terms of whether spouses' changes in fat intake over six months predict their own weight loss, this effect was significant, *β* = .36, *p* = .006. Specifically, spouses who decreased their fat intake had higher percent weight loss. 

**Figure 3:**
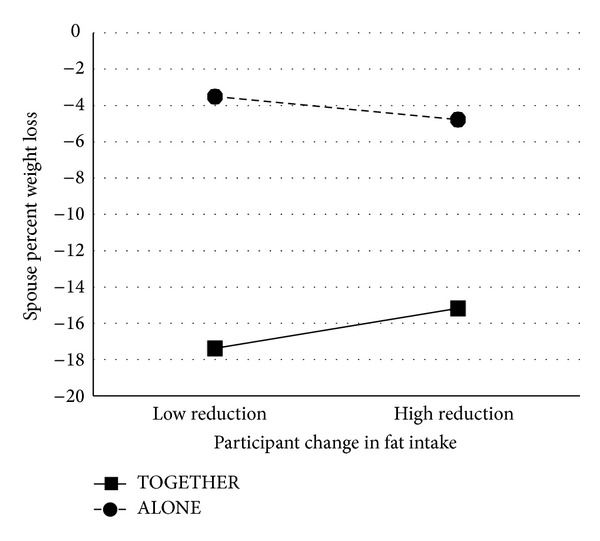
Interaction between treatment group and participants' fat intake change when predicting spousal weight loss. High and low fat intake reduction are defined as ±1 SD from the mean reduction score of 5.82% kcal. “High reduction” thus corresponds to a reduction of 15.14% kcal, and “low reduction” corresponds to a gain of 3.50% kcal.

 In terms of whether participants' changes in fat intake predict their spouses' weight loss, we found that spouses had higher percent weight loss when their participants did not decrease their fat intake as much, *β* = −.21, *p* = .037. However, this effect depended on which treatment group the couple participated in, *β* = .20, *p* = .038 (see Figure 3). We found a similar pattern of results to that of energy intake. The effect of treatment group on weight loss was greater for spouses who were coupled with a participant who did not reduce their own fat intake as much. Specifically, those spouses had higher percent weight loss when treated in the TOGETHER group, but lower percent weight loss when they were untreated in the ALONE group. When participants did have larger changes in fat intake, the effect of treatment group on their spouses' weight loss was not as large. In this case, the weight losses of spouses in the ALONE group benefitted from their partner's changes in fat intake, whereas the weight losses of spouses in the TOGETHER group did not. 

### 3.3. Physical Activity

 The relationship between six-month changes in physical activity (total exercise minutes/week) and weight loss was examined using APIM. No effects were found for our four primary research questions examining the impact of couple members' behavior changes on self or other couple member. Other than the treatment group predicting the weight loss of participants and spouses, no effects were significant.

## 4. Discussion

 To the best of our knowledge, this is the first study to explore the relationships between spouses' behavior changes and one another's weight loss, an important step at elucidating how spouses can be effectively incorporated into obesity treatment. We found overall that the weight loss intervention itself was the best predictor of weight loss; however, our four primary research questions more specifically examined the relationships between spouses' weight and behavior changes. 

 Most notable were our results that diet changes made by participants predicted their spouses' weight loss. This effect, however, depended on the treatment group in which the couple was enrolled; the weight losses of spouses in the ALONE condition were particularly dependent on their partners' changes in diet behaviors, suggesting a ripple effect from treated participants to their untreated partners. This pattern held true for changes in energy and fat intake, but not for physical activity. Further, the participant-spouse correlations for caloric intake and BMI were more robust in the ALONE condition at the end of the intervention. We speculate that compared to TOGETHER spouses who may have been more influenced by changes made to their home environments, ALONE spouses who did not receive any active intervention may have been more reliant on their participant's diet changes because those were the only changes to which they were exposed. 

 These findings are consistent with research that suggests that weight loss interventions have a beneficial impact on untreated spouses in research [2, 35] and clinical [36] settings. Across these studies changes in diet were associated with weight losses, and it was speculated that ripple effects were due to dietary modifications [2, 35, 36]. Our study demonstrates the participant's dietary changes were related to untreated spouses' weight losses, consistent with behavioral theory. Accordingly, spouses in the ALONE condition may have benefitted because the participants made weight loss-promoting changes to the shared home environment. There may have been greater availability of lower calorie drinks [37], fewer food choices [38], and smaller meals [39], all of which have been found to benefit weight loss. Further, the participants' changes in physical activity may not have been related because cues in this domain may be harder to spread in the shared home environment. Modest changes in the physical activity also may have reduced our ability to detect reciprocal impacts on weight loss.

 When we examined whether spouses' behavior changes impacted the participants' weight loss, we found no reverse effect. These findings were predicted for participants enrolled in the ALONE condition. More unexpectedly, the spousal social network did not provide benefits above and beyond the active weight-loss program to participants in couples that participated in the TOGETHER condition. While these spouses lost more weight overall than those enrolled in the ALONE condition, they had greater weight losses when their partners had lesser reductions in caloric intake and fat. These results may be because changes in the shared home environment were already being made as a result of being in this treatment condition, and there was no added value of behavior changes made by spouses. Perhaps when participants were not making as many changes, their spouses took on a more agenetic role in the intervention. They may have changed their own behaviors or home environment more to support their lesser-performing participants, especially because they also received weight-loss training and knew they were ultimately there to support the participants. How to enlist spouses in weight-loss treatment so that both partners receive maximum benefit remains an open question in the field. 

 We also examined the impact of the participants' or spouses' behavior changes on their own weight loss as a point of comparison. Our results did not show that participant's diet or activity changes were related to their own weight loss; instead, participants' weight loss was primarily predicted by group membership. We found, conversely, that spouse's diet changes predicted their own weight loss. 

 These findings are inconsistent with much of the existing literature and we speculate that they may be due to design and selection effects. The home environment intervention included many manipulations to the physical and social environment and it is likely we did not isolate its impact to the behaviors assessed in this secondary data analysis, despite finding that group membership predicted weight loss. Specifically, the participant who enrolled in the study was likely highly motivated, thus their energy intake and expenditure may have been impacted by multiple motivational and behavioral factors. The weight loss of spouses, however, may have been more directly influenced by intervention effects, including the dietary changes in the home environment from participating in the intervention (i.e., TOGETHER spouses) or from the home environment changes made by their spouse who participated intervention (i.e., ALONE spouses). Because we examined each behavior in isolation we were unable to detect the complex impact of the home environment intervention. It is likely that if we examined all of these behaviors as predictors simultaneously, we may have found that their combined effect is weight loss.

 Additional limitations of this study include the use of self-report measures for diet and activity behaviors. Use of these measurement tools may have led to biased or imprecise intake and expenditure estimates impacting our analyses of weight loss. Increased measurement error makes effects more difficult to detect, so it is possible that the effects we found in the current study would be stronger if it were to be replicated with more objective measurement. Additionally, we focused exclusively on variables related to behavior and weight change in order to make a first step toward understanding spousal processes in weight loss. In order to further elucidate the impact of the shared spousal environment, future studies would benefit from including variables of the home food and physical activity environment as well as of the spousal relationship. An additional concern is that spouses included in the study were willing to enter weight-loss treatment, thereby creating a more motivated or supportive sample that may not generalize to nontreatment seeking couples. Perhaps these spouses were more easily influenced by participants than would those who would not seek treatment. Future studies should aim for a more diverse sample. 

## 5. Conclusions

 Our findings that dietary changes ripple across spousal dyads to benefit weight loss in untreated partners provide further support that behavioral weight-loss interventions have an impact beyond the individual. Existing programs that focus their assessments exclusively on the individual may not be measuring the full impact of intervention benefits, particularly regarding social networks like marriage. Our unique contribution is identifying the powerful pathway of dietary changes in impacting weight loss in untreated partners. Additional research should determine how best to harness the spousal relationships to benefit weight loss of partners enrolled in direct treatment. Future studies should evaluate relationship dynamics amongst couples that might impact their success to effect change in one another. 

## Figures and Tables

**Table 1:** Demographics of participants and spouses, mean (SE), %.

	Participants		Spouses	
	ALONE	TOGETHER	ALONE	TOGETHER
	*N* = 68	*N* = 64	*N* = 68	*N* = 64
Age	51.2 (1.00)	50.73 (1.22)	52.49 (1.08)	50.72 (1.20)
Gender (% female)	72.10%	71.90%	27.90%	28.10%
Ethnicity (% Caucasian)	89.70%	98.40%	89.70%	93.80%
BMI	35.70 (0.70)	36.38 (0.77)	32.58 (0.62)	32.46 (0.71)

**Table 2:** Weight and behavior changes by treatment group, mean (SD).

Variable	Overall	ALONE	TOGETHER	*p*
Participant				
Weight (kg)				
Baseline	101.73 (22.55)	101.64 (22.15)	101.83 (23.14)	.96
Δ Baseline to 6 months	−9.13 (8.01)	−8.22 (8.40)	−10.06 (7.56)	.19
Energy intake (kcal/day)				
Baseline	2010.12 (846.22)	1968.69 (776.79)	2054.11 (918.41)	.56
Δ Baseline to 6 months	−546.62 (748.52)	−378.49 (666.35)	−690.72 (789.95)	.033
Fat intake (% kcal/day)				
Baseline	39.24 (7.61)	38.75 (7.03)	39.77 (8.21)	.44
Δ Baseline to 6 months	−5.82 (9.32)	−3.51 (6.85)	−7.74 (10.64)	.014
Physical activity (minutes/week)				
Baseline	57.58 (149.57)	44.56 (146.79)	71.41 (152.41)	.31
Δ Baseline to 6 months	47.81 (239.76)	48.73 (235.95)	46.94 (245.25)	.97

Spouse				
Weight (kg)				
Baseline	97.80 (21.02)	99.01 (19.70)	96.51 (22.43)	.50
Δ Baseline to 6 months	−7.37 (6.84)	−3.77 (5.27)	−10.91 (6.35)	<.001
Energy intake (kcal/day)				
Baseline	1982.60 (984.10)	2032.06 (1018.37)	1930.05 (951.53)	.55
Δ Baseline to 6 months	−396.01 (893.61)	−319.14 (1044.07)	−458.30 (753.95)	.43
Fat intake (% kcal/day)				
Baseline	38.33 (7.71)	39.05 (6.58)	37.56 (8.74)	.27
Δ Baseline to 6 months	−2.74 (8.47)	−0.32 (7.65)	−4.71 (8.65)	.007
Physical activity (minutes/week)				
Baseline	61.27 (147.06)	44.74 (107.82)	78.83 (178.88)	.19
Δ Baseline to 6 months	32.77 (204.85)	21.75 (222.35)	42.50 (189.42)	.59

**Table 3:** Correlations between spouses' behaviors by treatment group at baseline, 6 months, and change over 6 months.

Variable	Overall	TOGETHER	ALONE
BMI (kg/m^2^)			
Baseline	.218*	.212	.229
6 months	.225*	.145	.321*
Δ Baseline to 6 months	.480**	.554**	.420**
Physical activity (minutes/week)			
Baseline	.198*	.241	.120
6 months	.102	.111	.073
Δ Baseline to 6 months	.128	.146	.112
Energy intake (kcal/day)			
Baseline	.251**	.262*	.251*
6 months	.208*	.009	.313*
Δ Baseline to 6 months	.187	.177	.193
Fat intake (% kcal/day)			
Baseline	.344**	.436**	.233
6 months	.531**	.642**	.285
Δ Baseline to 6 months	.520**	.528**	.423**

*p < .05,  **p < .01.
